# Socio-Ecological Variables Associated with Context-Specific Sitting Time in Belgian Older Adults: A One-Year Follow-Up Study

**DOI:** 10.1371/journal.pone.0167881

**Published:** 2016-12-20

**Authors:** Cedric Busschaert, Anne-Lore Scherrens, Ilse De Bourdeaudhuij, Greet Cardon, Jelle Van Cauwenberg, Katrien De Cocker

**Affiliations:** 1 Department Movement & Sport Sciences, Ghent University, Ghent, Belgium; 2 Research Foundation Flanders (FWO), Brussels, Belgium; 3 Department Public Health, Ghent University, Ghent, Belgium; 4 Department Primary Care, Vrije Universiteit Brussel, Brussels, Belgium; 5 Department of Human Biometry and Biomechanics, Vrije Universiteit Brussel, Brussels, Belgium; Universidad Europea de Madrid, SPAIN

## Abstract

**Introduction:**

Knowledge about variables associated with context-specific sitting time in older adults is limited. Therefore, this study explored cross-sectional and longitudinal associations of socio-demographic, social-cognitive, physical-environmental and health-related variables with sitting during TV viewing, computer use and motorized transport in older adults.

**Methods:**

A sample of Belgian older adults completed structured interviews on context-specific sitting time and associated variables using a longitudinal study design. Objective measurements of grip strength and physical performance were also completed. Complete baseline data were available of 258 participants (73.98±6.16 years) of which 229 participants remained in the study at one year follow-up (retention rate: 91.60%). Cross-sectional correlates (baseline data) and longitudinal predictors (change-scores in relation with change in sitting time) were explored through multiple linear regression analyses.

**Results:**

Per context-specific sitting time, most of the cross-sectional correlates differed from the longitudinal predictors. Increases over time in enjoyment of watching TV (+one unit), encouragement of partner to watch less TV (+one unit) and TV time of partner (+30.0 min/day) were associated with respectively 9.1 min/day (p<0.001), 16.0 min/day (p<0.001) and 12.0 min/day (p<0.001) more sitting during TV viewing at follow-up. Increases over time in enjoyment of using a computer (+one unit), the number of smartphones and tablets (+1) and computer use of the partner (+30.0 min/day) were associated with respectively 5.5 min/day (p < .01), 10.4 min/day (p < .05) and 3.0 min/day (p < .05) more sitting during computer use at follow-up. An increase over time in self-efficacy regarding taking a bicycle or walking was associated with 2.9 min/day (p < .05) less sitting during motorized transport at follow-up.

**Conclusions:**

The results stressed the importance of looking at separate contexts of sitting. Further, the results highlighted the importance of longitudinal research in order to reveal which changes in particular variables predicted changes in context-specific sitting time. Variables at the social-cognitive level were most frequently related to context-specific sitting.

## Introduction

Older adults are subject to high levels of total sedentary behaviour, which is defined as any waking activity characterized by an energy expenditure ≤ 1.5 metabolic equivalents (METs) performed in a sitting/reclining posture [1]. According to a recent review, objective measurements of sedentary behaviour showed that older adults sit on average 9.4 hours/day [2]. Sitting receives increasing attention in terms of chronic disease prevention [3], as it has been identified as an independent risk factor, for all-cause mortality, metabolic syndrome, waist circumference, overweight/obesity, increased risk of depression and poor bone health in older adults [4–6]. For example, it was found that sitting during TV viewing was positively associated with waist circumference among UK older adults [7]. Further, TV viewing was also negatively associated with leg and total body lean mass among community-dwelling older adults [8]. Also, a meta-analysis revealed that prolonged sitting during TV viewing (pooled relative risk: 1.13) and computer or internet use (pooled relative risk: 1.22) were associated with depression [9]. Also, evidence suggested to avoid long periods of uninterrupted sitting time, as more breaks in sitting time were associated with lower triglycerides, waist circumference, Body Mass Index (BMI), and 2-h plasma glucose in adults [10]. Next, replacing sitting time by standing may be beneficial for cardio-metabolic health [11]. However, more research is still needed to explore how many times long periods of sitting should be interrupted (i.e. number of breaks per hour) together with the minimum duration and/or intensity of the break that may be necessary for positive health effects [10].

Sitting is a complex behaviour that occurs in multiple contexts, e.g. TV viewing, computer use and motorized transport [12–14]. Additionally, evidence suggested that correlates (cross-sectional) and predictors (longitudinal) might be context-specific, however, more research is warranted to map (differences between) context-specific correlates and predictors in older adults [15]. Finally, focusing on identifying correlates/predictors of context-specific sitting time will be of added value for developing more-effective interventions in the future, as gathering associated variables of total sitting time is too broad to target the complexity of sitting time. Future interventions will be more effective when targeting particular contexts of sitting time. Owen et al. [13] indicated the importance of investigating associated variables of sitting time in particular contexts, as this may lead to more-effective interventions in the future. However, it may be advised to not set up interventions that solely focus on one context of sitting time, as this may not cause meaningful changes in total sitting time.

Socio-ecological models are commonly used to explain sitting time and state that multiple levels of variables influence context-specific sitting time [13]. Owen et al. [13] stressed the need for future research to identify associated variables at multiple levels, including individual, social, organizational/community, environmental and policy levels. Still, knowledge about these variables is limited in older adults compared to other age groups [16, 15]. A systematic review of Chastin et al. [15] within the DEterminants of DIet and Physical ACtivity (DEDIPAC) knowledge hub revealed that evidence on correlates of sitting time in older adults was mostly gathered using cross-sectional studies (n = 22) of which the focus was on *leisure time sitting*, *TV viewing*, *leisure screen time* and *sitting in cars*. Important to notice is the dearth of information from longitudinal studies investigating associated variables of sitting time which highlight the limited evidence of causal relationships among older adults [15]. In older adults, more time is spent sedentary as age advances [2] and (sedentary) behaviours can change in a relatively short amount of time due to sudden changes in health conditions (becoming ill for example) or living situations (loss of the partner for example. To our knowledge, only three studies previously investigated longitudinal associations with sitting time in this age group [17–19]. These three studies only focused on total sitting time or TV viewing. In addition, Chastin et al. [15] highlighted that mostly non-modifiable variables (e.g. socio-demographics) were investigated as potential associated variables of sitting time, which stresses the need for additional research on (multilevel) modifiable variables (e.g. environmental and social level) to inform intervention developers targeting sitting time. Therefore, it is advised to explore motivational and contextual associated variables of sitting time [20]. Furthermore, particularly in older adults, the inclusion of health indicators (pain, fear of falling) as potential variables associated with sitting time is rare, but important [15, 21].

Summarized, it is needed to further explore cross-sectional and longitudinal multilevel variables associated with context-specific sitting time in older adults. The objective of the present longitudinal study with a follow-up period of one year was to investigate multilevel correlates and predictors of context-specific sitting time in older adults who still live independently or in a service flat. First, cross-sectional correlates (i.e. socio-demographic, social-cognitive, physical environmental and health-related variables) of Belgian older adults’ context-specific sitting time at baseline (i.e. TV-viewing, computer use and motorized transport) were identified (aim a). Secondly, it was examined if changes in these socio-demographic, social-cognitive, physical environmental and health-related variables from baseline to follow-up predicted changes in older adults’ context-specific sitting (aim b).

## Materials and Methods

### Study design

The present study was a longitudinal study with a follow-up period of one year. Baseline data was gathered between September 2013 and April 2014, follow-up data between September 2014 and May 2015.

### Subjects and procedures

Older adults’ contact information (full name, address and date of birth of potential participants aged 65 years or older) was gathered via the public service department of the city Sint-Niklaas (metropolitan city with approximately 73,000 inhabitants in Flanders, northern Dutch-speaking part of Belgium). An information letter (background, objectives, practical information about the study, and the announcement of a telephone call from a researcher or visit during the following days) was sent to a random sample of 961 older adults at baseline. From the 961 potential participants, 919 older adults actually received the information letter (42 persons were not reached of which 18 older adults deceased and 24 older adults had moved to another address). After sending the information letter, potential participants were contacted by telephone. The researchers tried to contact the older adults up to three times on different days and different moments during the day [22]. Participants who agreed to participate in the present study completed a questionnaire through a structured interview led by a trained researcher at the participants’ homes [23]. To be included in the study, participants had to be 65 years or older, living independently or in a service flat, Dutch-speaking and be able to stand up (e.g. no recent surgery or other medical condition in which a person cannot stand up). Six hundred sixty-one older adults were not included in the analyses, due to several reasons: not present during the three attempts to contact him/her (n = 59); refused to participate (n = 567); not able to participate due to illness (n = 30); not Dutch-speaking (n = 4); not able to stand up (n = 1). This resulted in a final sample of 258 older adults at baseline (participation rate: 28.07%).

At follow-up, all baseline participants received an information letter of the follow-up measurement (same methodology was used as during baseline). From the 258 participants at baseline, 250 older adults actually received the information letter (7 older adults deceased prior to the follow-up measurement, one moved to another address). Twenty-one older adults did not participate at the follow-up measurement due to several reasons (i.e. not present during the three attempts to contact him/her: n = 1; refused to participate: n = 16; not able to participate due to illness: n = 4). This resulted in a final sample of 229 older adults participating both at baseline and follow-up (retention rate: 91.60%).

Possible differences between the older adults who dropped out and the older adults who remained in the study were analysed by using drop-out analyses. Older adults who dropped out were older (77.55 ± 8.43 years vs. 73.53 ± 5.68 years, p = 0.018) than older adults who remained in the study. No differences were found for BMI (p = 0.280), sex (p = 0.505) and educational level (p = 0.401). Both at baseline and follow-up, participants provided written informed consent. The study protocol was approved by the Ghent University Hospital Ethics Committee.

### Measures

In the present study a questionnaire was used that was specifically developed for older adults [23]. This questionnaire assessed total sitting time, context-specific sitting time and its potential socio-demographic, social-cognitive, physical environmental and health-related correlates or predictors. The validity of this questionnaire has been evaluated by comparing the self-reported sitting time to activPAL data and revealed moderate-to-good validity for total sitting time on an average day (ρ = 0.48) [23]. The questionnaire also showed good test-retest reliability (Intraclass Correlation Coefficients (ICC) = 0.80) for total sitting time on an average day [23]. Additionally, every context-specific sitting time (i.e. TV viewing, computer use and motorized transport) included in the present study, showed acceptable-to-good test-retest reliability (ICC = 0.48–0.83) [23]. Furthermore, the test-retest reliability of the included potential predictors of context-specific sitting time was measured and revealed moderate-to-excellent results [23]. However, particular social-cognitive variables, which revealed poor reliability, were excluded from the present analyses (indicated in Table 1).

**Table 1:** Overview of the included item-specific social-cognitive variables, their mean values at baseline and follow-up and the mean change score.

	Item questionnaire	Baseline: mean (SD)	Follow-up: mean (SD)	Change-score follow-up—baseline: mean (SD)
**TV viewing**				
Attitude 1^(a)^	I think watching TV is pleasant	3.99 (0.94)	4.06 (1.01)	0.08 (1.06)
Attitude 2^(a)^	Watching TV takes time away from doing other important things	1.66 (1.22)	1.60 (1.20)	-0.07 (1.37)
Attitude 3^(a)^	I enjoy watching TV for many hours at a time	2.52 (1.61)	2.91 (1.70)	0.37 (1.71)
Attitude 4^(a)^	Watching TV is my way to relax	3.31 (1.46)	3.48 (1.56)	0.18 (1.61)
Self-efficacy 1^(a)^	I consider it possible to reduce my TV time	2.45 (1.66)	2.66 (1.70)	0.16 (2.03)
Self-efficacy 2^(a)^	I consider it possible to turn off the TV during weekend days until 5:00 p.m.	4.36 (1.28)	4.37 (1.32)	-0.07 (1.45)
Self-efficacy 3^(a)^	I consider it possible to turn off the TV during meals	4.50 (1.16)	4.49 (1.24)	-0.04 (1.22)
Norm^(a)^	I think that I spend too much time watching TV(*)	/	/	/
Social norm^(a)^	My partner thinks I spend too much time watching TV	1.47 (1.16)	1.57 (1.16)	0.12 (1.37)
Social support 1^(a)^	My partner encourages me to watch less TV	1.30 (0.94)	1.50 (1.15)	0.23 (1.26)
Social support 2^(a)^	My friends encourage me to watch less TV	1.10 (0.42)	1.15 (0.59)	0.05 (0.65)
Modelling ^(b)^	How long, on average, does your partner spend watching TV?	203.34 (89.32)	211.47 (91.45)	9.26 (82.32)
**PC-use**				
Attitude 1^(a)^	I think using a computer is pleasant	4.34 (1.03)	4.35 (1.13)	0.06 (1.04)
Attitude 2^(a)^	Using a computer takes time away from doing other important things	1.45 (0.98)	1.22 (0.71)	-0.24 (1.04)
Attitude 3^(a)^	I enjoy using a computer for many hours at a time	2.20 (1.47)	2.18 (1.48)	0.09 (1.54)
Attitude 4^(a)^	Using a computer is my way to relax	2.86 (1.69)	2.80 (1.64)	0.01 (1.43)
Self-efficacy 1^(a)^	I consider it possible that I do not use a computer for some days in the week	3.90 (1.57)	3.45 (1.74)	-0.42 (1.99)
Self-efficacy 2^(a)^	I consider it possible to reduce my computer time(*)	/	/	/
Norm^(a)^	I think that I spend too much time using a computer	1.35 (0.91)	1.38 (1.02)	0.06 (1.07)
Social norm^(a)^	My partner thinks I spend too much time using a computer	1.48 (1.18)	1.53 (1.19)	0.05 (1.15)
Social support 1^(a)^	My partner encourages me to spend less time using a computer	1.20 (0.77)	1.48 (1.13)	0.32 (1.13)
Social support 2^(a)^	My friends encourage me to spend less time using a computer	1.04 (0.26)	1.07 (0.28)	0.02 (0.39)
Modelling ^(b)^	How long, on average, does your partner sit when using the computer (tablet, internet on smartphone, laptop, desktop,…)?	69.81 (103.92)	84.23 (97.16)	10.18 (82.35)
**Motorized transport**				
Attitude 1^(a)^	I think using motorized transport is pleasant	3.92 (1.38)	3.89 (1.41)	-0.06 (1.52)
Attitude 2^(a)^	I think it is pleasant to work or to rest as a passenger during motorized transport(*)	/	/	/
Attitude 3^(a)^	I feel lazy arriving at my destination after motorized transport^(^)^	4.66 (0.88)	4.65 (0.98)	-0.01 (0.97)
Self-efficacy 1^(a)^	I consider it possible to get off the bus/metro spontaneously 1 stop before my destination and to walk the remaining distance	2.96 (1.84)	3.01 (1.77)	-0.07 (2.00)
Self-efficacy 2^(a)^	I consider it possible to park the car somewhat further spontaneously and to walk the remaining distance	3.10 (1.84)	3.42 (1.78)	0.17 (1.93)
Self-efficacy 3^(a)^	I consider it possible to take the bicycle or to go by foot spontaneously even if it is possible to use a car	3.96 (1.57)	4.04 (1.60)	-0.04 (1.44)
Norm^(a)^	I think that I spend too much time using motorized transport	1.55 (1.18)	1.42 (1.08)	-0.14 (0.96)
Social norm^(a)^	My partner thinks I spend too much time using motorized transport	1.20 (0.71)	1.35 (1.02)	0.09 (0.90)
Social support 1^(a)^	My partner encourages me to use (more often) active transport (to bicycle or to walk)	2.26 (1.69)	2.48 (1.76)	0.16 (1.95)
Social support 2^(a)^	My friends encourage me to use (more often) active transport (to bicycle or to walk) (*)	/	/	/
Modelling ^(c)^	The most chosen transportation possibility from my partner	0.47 (0.50)	0.47 (0.50)	-0.02 (0.40)

Note:

(*) indicates an item that is not included due to low test-retest reliability.

(^) indicates an item that was recoded because of negative scoring. Abbreviations: PC-use (computer use), TV (television), SD (standard deviation).

Answering categories^(a)^: strongly disagree; somewhat disagree; neutral; somewhat agree; strongly agree [1–5]

Answering categories^(b)^: 0 min/day; 7.5 min/day; 22.5 min/day; 45 min/day; 90 min/day; 150 min/day; 210 min/day; 270 min/day; 330 min/day; 390 min/day; 450 min/day

Answering categories^(c)^: motorized transport; active transport (walking, bicycling) [0;1]

#### Context-specific sitting time

Three context-specific sitting times (i.e. *TV viewing*, *computer use* and *motorized transport*) were measured identically at baseline and follow-up and were assessed by questioning sitting time in the past seven days (weekday and weekend day separately). The answer categories for sitting during TV viewing and computer use were identical: ‘none’, ‘1–15 min/day’, ‘15–30 min/day’, ‘30–60 min/day’, ‘1–2 hours/day’, ‘2–3 hours/day’, ‘3–4 hours/day’, ‘4–5 hours/day’, ‘5–6 hours/day’, ‘6–7 hours/day’ or ‘more than 7 hours/day’. For sitting during motorized transport, the answer categories were slightly different (‘none’, ‘1–15 min/day’, ‘15–30 min/day’, ‘30–45 min/day’, ‘45–60 min/day’, ‘60–90 min/day’, ‘90–120 min/day’, ‘2–2.5 hours/day’, ‘2.5–3 hours/day’, ‘3–4 hours/day’, ‘4–5 hours/day’, ‘5–6 hours/day’, ‘6–7 hours/day’ or ‘more than 7 hours/day’).

#### Correlates of context-specific sitting time

All included correlates of context-specific sitting were measured identically at baseline and follow-up.

Socio-demographic variables. The socio-demographic variables that were assessed were: *educational level*, *sex*, *family situation*, *age* (determined based on date of birth and date of filling out questionnaire) and *residential area* (detailed information in Table 2).

**Table 2:** Overview of the included socio-demographic variables and the recoding used for the cross-sectional and longitudinal analyses.

	Questionnaire item	Original answer category	Recoding used for cross-sectional analyses		Recoding used for longitudinal analyses	
			Original category	Recoded variable: [name, proportion]	Changes from baseline to follow-up	Recoded variable: [name, proportion]
Family situation	How would you describe your family situation?	1 = single; 2 = having a partner, but living independently; 3 = living with a partner; 4 = married; 5 = widow/widower	1 / 2 / 5	0 [not living with a partner, 32.3%]	no changes, 4 changed into 3, 2 changed into 1, 1 changed into 2, or 3 changed into 4	0 [stable, 94.7%]
					1 or 2 changed into 3 or 4	1 [start living together, 0.9%]
			3 / 4	1 [living with a partner, 67.7%]	3 or 4 changed into 1 or 2 or 5	2 [start living alone, 4.4%]
Educational level	What is your highest achieved diploma or certificate?	1 = primary school; 2 = secondary education; 3 = higher education, non-university; 4 = university	1 / 2	0 [low educational level, 75.7%]	--	**-**
			3 / 4	1 [high educational level, 24.3%]		
Sex	What is your sex?	1 = male; 2 = female	1	1 [male, 47.3%]	--	**-**
			2	0 [female, 52.7%]		
Residential area	In which type of area do you live?	1 = countryside; 2 = village or town; 3 = cities suburbs; 4 = city	1 / 2	0 [countryside, village, town, 27.2%]	--	**-**
			3 / 4	1 [cities suburbs and city, 72.8%]		

Note: “-” indicates variables that were not included in longitudinal analyses.

Social-cognitive variables. The social-cognitive variables (i.e. *attitude*, *self-efficacy*, *norm*, *social norm*, *social support* and *modelling*) were assessed for sitting during TV viewing, computer use and motorized transport. Importantly, the social-cognitive variables were included as context- and item-specific variables (e.g. attitude 3 TV viewing: ‘I enjoy watching TV for many hours at a time’; attitude 4 TV viewing: ‘watching TV is my way to relax’), as this ensured more detailed information (see Table 1) [24]. The answer categories of the variables measuring attitude, (social) norm, self-efficacy and social support ranged from ‘strongly disagree’ to ‘strongly agree’ (five-point Likert scale). The answer categories concerning modelling (partner) for TV viewing and computer use ranged from ‘none (no TV viewing or computer use)’ to ‘more than 7 hours/day’ (11-point Likert scale). For motorized transport, a dichotomous answer category was used (i.e. active transport or motorized transport).

Physical environmental variables. For each context of sitting time identical physical environmental variables were assessed both at baseline and follow-up. Participants were asked to register the number of equipment regarding TV viewing and computer use available in the household that they operate themselves. For TV-viewing, the variable ‘*TV set*’ was calculated by summing the number of televisions and the variable ‘*other TV-viewing equipment*’ by summing the number of laptops, desktops, smartphones and tablets. For computer use, the variable ‘*computer equipment*’ was calculated by summing the number of laptops and desktops. The variable ‘*other equipment for computer use*’ was obtained by summing the number of smartphones and tablets. The answer categories for both equipment regarding TV-viewing and computer use ranged from ‘none’ to ‘more than 5’ (7-point Likert scale). Two additional physical environmental variables were included in the analyses regarding TV viewing. Participants had to rate the items ‘*the remote controller (TV) can always be found closely to me when I need it*’ and ‘*the couches at our place are comfortable to sit for a long time*’, with answer categories ranging from ‘strongly disagree’ to ‘strongly agree’ (five-point Likert scale). For motorized transport, older adults documented the *number of all operational motorized vehicles* (no electric bicycles) available in the household. A detailed overview of the included physical environmental variables can be found in Table 3.

**Table 3:** Overview of the included physical environmental variables, their mean values at baseline and follow-up and the mean change scores.

	Items	Baseline: mean (SD)	Follow-up: mean (SD)	Change-scorefollow-up—baseline: mean (SD)
TV set	How many TV’s do you use and are present at your home?^(1)^	1.29 (0.54)	1.26 (0.52)	-0.02 (0.32)
Other TV-viewing equipment	How many of the following electronic devices do you use and are present at your home?			
	a) laptops^(1)^; b) desktops^(1)^; c) smartphones^(1)^; d) tablets^(1)^	0.97 (1.09)	1.10 (1.22)	0.08 (0.80)
Remote controller	The remote controller (TV) can always be found closely to me when I need it^(2)^	4.70 (0.85)	4.58 (1.06)	-0.12 (1.25)
Sitting furniture (couches)	The couches at our place are comfortable to sit for a long time^(2)^	4.73 (0.77)	4.77 (0.66)	-0.02 (0.82)
PC equipment	How many of the following electronic devices do you use and are present at your home?			
	a) laptops^(1)^; b) desktops^(1)^	0.75 (0.82)	0.75 (0.74)	-0.05 (0.59)
Other equipment for computer use	How many of the following electronic devices do you use and are present at your home?			
	a) smartphones^(1)^; b) tablets^(1)^	0.22 (0.50)	0.35 (0.71)	0.13 (0.54)
Motorized vehicles	How many operational motorized vehicles (no electric bicycles) are there present in the household, even the ones you do not use yourself?^(3)^	1.04 (0.70)	1.09 (0.78)	0.02 (0.38)

Note: PC (computer), TV (television), SD (standard deviation).

Answering categories^(1)^: ‘none’, ‘1’, ‘2’, ‘3’, ‘4’, ‘5’ or ‘more than 5’ [0–6]

Answering categories^(2)^: ‘strongly disagree’, ‘somewhat disagree’, ‘neutral’, ‘somewhat agree’, ‘strongly agree’ [1–5]

Answering categories^(3)^: open-ended question

Health-related variables. Different health-related variables were assessed using objective (by a trained researcher after filling out questionnaire) or subjective measurements (part of the questionnaire). Older adults rated the following items (five-point Likert scale): *general health* (‘poor’ to ‘excellent’), to what extent *pain* interfere with their activities during the past weeks (‘not at all’ to ‘quite a lot’) and *fear of falling* (‘none of the time’ to ‘all of the time’) [25–27]. Participants’ *weight* up to 0.1 kg (SECA 813 Robusta weight scale) and *height* up to 0.1 cm (SECA 213 stadiometer) was measured in order to calculate BMI (kg/m^2^). Further, *grip strength* (kg) of participants was measured twice, of which the best was included in the analyses, by using a hand dynamometer (Hydraulic Saehan). Participants were explained to stand upright, holding the dynamometer in their dominant hand with the arm held out downwards (without making contact with the body). The ‘Short Physical Performance Battery’ (SPPB) was used to measure the *physical performance*, consisting of three tests: balance, 3.0 meter walk (instead of 2.4 meter) and a chair stands test [28]. Afterwards, every test was scored (0–4) individually with ‘0’ indicating that the person was not capable or did not perform the test and ‘4’ for the best score, resulting in a final SPPB score (maximum of 12 points). A detailed overview of the included health-related variables can be found in Table 4.

**Table 4:** Overview of the included health-related variables, their mean values at baseline and follow-up and the mean change scores.

	Items	Baseline mean (SD)	Follow-up: mean (SD)	Change-score follow-up—baseline: mean (SD)
General health	In general, how would you rate your health?^(1)^	3.15 (0.83)	3.08 (0.80)	-0.11 (0.76)
Pain that interfere with activities	To what extent did pain interfere with your activities during the past weeks?^(2)^	1.93 (1.11)	1.81 (1.05)	-0.05 (1.09)
Fear of falling	How often do you have fear of falling?^(3)^	2.16 (1.28)	2.06 (1.27)	-0.10 (1.03)
BMI (kg/m^2^)	Objectively-measured height (cm) and weight (kg)	27.79 (4.01)	27.57 (4.09)	-0.31 (1.30)
Grip strength (kg)	Objectively-measured by using a hand dynamometer	26.17 (9.37)	26.02 (9.58)	-0.44 (4.22)
Physical performance	Short Physical Performance Battery^(4)^	10.03 (2.22)	10.35 (2.34)	0.12 (1.87)

Note: Abbreviations: BMI (Body Mass Index), SD (standard deviation).

Answering categories^(1)^: ‘poor’, ‘fair’, ‘good’, ‘very good’, ‘excellent’ [1–5]

Answering categories^(2)^: ‘not at all’, ‘a little bit’, ‘moderately’, ‘quite a bit’, ‘extremely’ [1–5]

Answering categories^(3)^: ‘none of the time’, ‘a little of the time’, ‘some of the time’, ‘most of the time’, ‘all of the time’ [1–5]

Scoring^(4)^: the Short Physical Performance Battery consists of three tests and every test was scored (0–4) individually with ‘0’ indicating that the person was not capable or did not perform the test and ‘4’ for the best score, resulting in a final score on the Short Physical Performance Battery of maximum 12 points.

#### Potential covariate of context-specific sitting time

The short-IPAQ (International Physical Activity Questionnaire) was used to estimate total physical activity (PA) at baseline and follow-up [29–31]. Participants reported the frequency (days/week) and time (hours or minutes per day) of their PA during the past seven days, including walking, moderate-intensity PA and vigorous-intensity PA.

### Data reduction

#### Context-specific sitting time

The midpoint values of the above-mentioned answer categories (i.e. **sitting time**) were determined in order to calculate a numeric variable for sitting during TV viewing, computer use and motorized transport. Subsequently, daily minutes for an average day were measured by using the following formula: ((sitting time on a weekday * 5) + (sitting time on a weekend day * 2))/7. Afterwards, baseline measurement was subtracted from follow-up measurement (i.e. follow-up minus baseline) to calculate a change-score for all included context-specific sitting times separately.

#### Correlates of context-specific sitting time

Potential cross-sectional correlates of sitting during TV viewing, computer use and motorized transport were identified by using the baseline measurements. Changes in socio-demographic, social-cognitive, physical environmental and health-related variables from baseline to follow-up were included as potential longitudinal predictors of changes in context-specific sitting times (subtraction of baseline measurements from follow-up measurements).

The following potential **socio-demographic** correlates were included in the baseline measurements: educational level, sex, family situation and residential area. In the longitudinal analyses, only a change-score was calculated for family situation, as other above-mentioned variables had a limited variance during one year of follow-up (i.e. residential area) or asking for a change was irrelevant (i.e. sex and educational level). Table 2 gives an overview of the included socio-demographic variables in the cross-sectional and longitudinal analyses (including scoring methods and descriptive statistics [% or mean ± SD]).

Furthermore, all **social-cognitive**, **physical environmental** and **health-related variables** were scored/recoded in the same direction to facilitate interpretation of the results (highest score is most positive answer). A change-score (i.e. follow-up minus baseline) for each of these variables was calculated. Importantly, item-specific values regarding the **social-cognitive variables** were used to calculate change-scores. More information on the scoring methods and descriptive statistics (mean ± SD) of the social-cognitive variables is provided in Table 1.

#### Potential covariate of context-specific sitting time

Walking, moderate-intensity PA and vigorous-intensity PA were processed using the scoring protocol from the IPAQ [32]. Total PA was measured by summing up the three PA variables. Total PA at baseline was included as a covariate in the cross-sectional analyses and a change-score for total PA (i.e. follow-up minus baseline) was included as a covariate in the longitudinal analyses.

### Statistical analyses

Statistical analyses for the cross-sectional and longitudinal research questions were performed in different software programs. First, for the cross-sectional analyses, potential correlates of context-specific sitting time at baseline (aim a) were examined using R Studio Version 0.98.507. Furthermore, SAS version 9.4 software (SAS institute Inc., Cary, NC, USA) was used to examine longitudinal changes in potential predictors related to changes in context-specific sitting time (aim b). Statistical significance was determined at α = 0.05.

#### Potential cross-sectional correlates of context-specific sitting time at baseline (aim a)

For the cross-sectional analyses, the statistical model used to examine the correlates of TV viewing was different than the models used for computer use and motorized transport due to different distributions of these dependent variables. TV viewing was normally distributed, so standard linear regression analyses were performed. Zero-inflated negative binomial regression models (ZINB) were used to examine the correlates of computer use and motorized transport, as these contexts were positively skewed and included many zero counts. The necessity of using zero-inflated models was confirmed by Vuong tests [33]. Based on Akaike’s Information Criterium (AIC) ZINB models were preferred over zero-inflated poisson (ZIP) models. The interpretation of ZINB models is two-fold as these models estimate simultaneously two regression coefficients per potential correlate. First, a logistic regression is fitted in which the relationship with the probability to be a person who did not sit during computer use or motorized transport is estimated (odds ratio (OR)). Simultaneously, ZINB models examine the relationship between potential correlates and the volume of computer use/motorized transport among those who did sit during computer use or motorized transport (negative binomial regression coefficient, expressed in minutes/week). The exponent of a negative binomial regression coefficient (expB) can be interpreted as a relative increase/decrease in sitting time during computer use or motorized transport associated with a one-unit increase in the correlate. For example, an expB of 1.60 for attitude implies that, among those who did sit during computer use, a one-unit higher score in attitude was associated with 60% more minutes/week in sitting during computer use.

Correlates of context-specific sitting time (i.e. TV viewing, computer use and motorized transport) were identified in four steps, separately for each included context-specific sitting time variable. First, multicollinearity between the quantitative correlates was tested per level (i.e. socio-demographic, social-cognitive, physical environmental and health-related variables). When two correlates belonging to one level showed Pearson correlation coefficients higher than 0.60 with each other [34], the correlate that had the lowest Pearson correlation coefficient with the context-specific sitting time variable was not included in the analyses. Following this procedure, ‘my partner encourages me to spend less time using a computer’ (social support 1 computer use) and ‘my partner encourages me to watch less TV’ (social support 1 TV viewing) were removed. Secondly, for each level, a model was fitted containing all correlates within that level (i.e. four regression models per context-specific sitting time variable). The correlates that showed p<0.10 with the context-specific sitting time variable were included in the next step [35, 36]. In step 3, multicollinearity between the quantitative correlates that yielded a p<0.10 in step 2 was tested. None of these correlates were highly correlated (Pearson r>0.60) with each other. In step 4, the correlates with p<0.10 in step 2 were combined into one model. The results of step 4 were presented and discussed in the current paper. All the cross-sectional analyses were adjusted for total PA at baseline.

#### Changes in potential predictors related to changes in context-specific sitting time (aim b)

For the longitudinal analyses, the change-scores for TV viewing, computer use and motorized transport were normally distributed and linear regression analyses were performed using PROC CALIS [37]. Full-information maximum likelihood was used as estimation procedure as this effectively utilizes all available information in the data. This procedure is recommended when dealing with missing data on both outcome and predictor variables, as was the case using the present longitudinal data [37]. The longitudinal analyses examined if change-scores of potential predictors predicted changes in context-specific sitting time. In these analyses the same stepwise approach was used as described above to identify the correlates. In step 1, no potential predictors were removed due to multicollinearity. Secondly, for each level separately, multiple regression models were performed containing all change-scores of potential predictors within that level (i.e. four regression models per context-specific sitting time variable). In step 3, none of the remaining change-scores of potential predictors were highly correlated with each other. In step 4, the change-scores of potential predictors with p<0.10 in step 2 were combined into one multiple linear regression model per context-specific sitting time variable. The results of step 4 were presented and discussed in the current paper. All the analyses were adjusted for change in total PA between baseline and follow-up and baseline context-specific sitting time. Furthermore the longitudinal analyses were adjusted for age at baseline, as older adults who dropped out were older than older adults who remained in the study.

## Results

### Sample characteristics

Table 5 gives an overview of the socio-demographic characteristics, BMI and context-specific sitting at baseline and follow-up.

**Table 5:** Sample characteristics at baseline and follow-up.

	BASELINE	FOLLOW-UP
Age (years, mean (SD))	73.98 (6.16)	74.58 (5.68)
Male gender (%)	47.30	48.20
High educational level (%)	24.30	27.30
BMI (kg/m^2^, mean (SD))	27.79 (4.01)	27.57 (4.09)
Family situation		
Married or living with partner (%)	67.70	65.00
Widow/widower (%)	18.30	23.60
Single (%)	11.70	10.50
Partner, but living apart (%)	2.30	0.90
TV viewing time (min/average day, mean (SD))	200.99 (98.29)	204.16 (94.74)
Computer use (min/average day, median; Q1-Q3)	7.50; 0.00–45.00	18.21; 0.00–45.00
Motorized transport (min/average day, median; Q1-Q3)	22.50; 9.62–28.93	26.79; 15.71–37.50

Note: Abbreviations: SD (standard deviation), BMI (Body Mass Index), min (minutes).

### Aim a: Cross-sectional analyses

The aim was to identify socio-demographic, social-cognitive, physical environmental and health-related correlates of sitting during TV viewing, computer use and motorized transport at baseline. The results of this aim are presented in Table 6.

**Table 6:** Associations between item-specific correlates and sitting during TV time, computer use and motorized transport: estimates, odds ratios, regression coefficients from the cross-sectional analyses using baseline data.

Correlates	Dependent variables									
	Sitting during TV viewing		Sitting during computer use				Sitting during motorized transport			
	Estimate (SE)	p	A) Logit model		B) Negative binomial model		A) Logit model		B) Negative binomial model	
			OR of being a person who did not sit during computer use^1^ (95% CI)	p	Regression coefficient: min/week [expB (95% CI)]	p	OR of being a person who did not sit during motorized transport^2^ (95% CI)	p	Regression coefficient: min/week [expB (95% CI)]	p
**Socio-demographic variables**										
Educational level	-12.97 (12.39)	ns	5.10 (0.19, 134.41)	ns	0.90 (0.65, 1.25)	ns	0.29 (0.002, 41.99)	ns	1.30 (0.98, 1.73)	ns
Sex	-	-	4.08 (0.21, 79.69)	ns	1.26 (0.83, 1.90)	ns	-	-	-	-
Family situation	-	-	-	-	-	-	-	-	-	-
Residential area	-	-	-	-	-	-	-	-	-	-
**Social-cognitive variables**										
Attitude 1	-	-	0.35 (0.12, 1.05)	ns	1.66 (1.43, 1.92)	***	-	-	-	-
Attitude 2	-	-	-	-	-	-	X	X	X	X
Attitude 3	12.73 (3.68)	***	-	-	-	-	-	-	-	-
Attitude 4	13.90 (4.03)	***	-	-	-	-	X	X	X	X
Self-efficacy 1	-	-	X	X	X	X	X	X	X	X
Self-efficacy 2	-11.60 (5.09)	*	X	X	X	X	X	X	X	X
Self-efficacy 3	-	-	X	X	X	X	-	-	-	-
Norm	X	X	-	-	-	-	0.16 (0.004, 6.01)	ns	1.14 (1.02, 1.28)	*
Social norm	16.33 (4.88)	**	-	-	-	-	5.65 (0.31, 103,00)	ns	1.23 (1.00, 1.50)	ns
Social support 1	X	X	X	X	X	X	-	-	-	-
Social support 2	-	-	X	X	X	X	X	X	X	X
Modelling	0.42 (0.06)	***	-	-	-	-	0.39 (0.03, 5.32)	ns	0.76 (0.58, 0.99)	*
**Physical environmental variables**										
TV set	-	-	X	X	X	X	X	X	X	X
Other TV-viewing equipment	-9.53 (4.79)	*	X	X	X	X	X	X	X	X
Remote controller	-7.49 (6.39)	ns	X	X	X	X	X	X	X	X
Sitting furniture (couches)	-	-	X	X	X	X	X	X	X	X
PC equipment (desktop & laptop)	X	X	0.25 (0.02, 2.74)	ns	1.13 (0.91, 1.41)	ns	X	X	X	X
Other equipment for computer use	X	X	0.09 (0.001, 8.91)	ns	1.22 (0.97, 1.53)	ns	X	X	X	X
Motorized vehicles	X	X	X	X	X	X	0.02 (0.001, 0.33)	**	1.17 (0.99, 1.39)	ns
**Health-related variables**										
General health	-16.26 (7.37)	*	-	-	-	-	-	-	-	-
Pain that interfere with activities	-	-	-	-	-	-	-	-	-	-
Fear of falling	-	-	-	-	-	-	-	-	-	-
BMI	2.98 (0.08)	*	-	-	-	-	-	-	-	-
Grip strength	-	-	0.74 (0.52, 1.05)	ns	1.02 (0.99, 1.04)	ns	-	-	-	-
Physical performance	-	-	1.06 (0.47, 2.39)	ns	1.09 (0.99, 1.20)	ns	-	-	-	-

Note: Potential correlates were identified by using baseline measurements. A thorough description of the included social-cognitive variables can be found in Table 1. For sitting during computer use and motorized transport, the interpretation of ZINB models is two-fold. First, a logistic regression is fitted in which the relationship with the probability to be a person who did not sit during computer use or motorized transport is estimated (logit model). Simultaneously, the exponent of a negative binomial regression coefficient (expB) can be interpreted as a relative increase (values >1) /decrease (values <1) in sitting time during computer use or motorized transport associated with a one-unit increase in the correlate among those who did sit during computer use or motorized transport.

^”1”^ number of zero counts = 119.

^“2”^ number of zero counts = 17.

For sitting during TV viewing, estimates can be interpreted as change in minutes/day, in which positive values indicate an increase in sitting during TV viewing and negative values indicate a decrease in sitting during TV viewing (expressed in minutes/day).

“X” indicates correlates not inserted in analyses for context-specific sitting time (i.e. low test-retest reliability, not measured for particular context or not inserted in analysis for particular context).

“-” indicates correlates that showed levels of significance p ≥ .10 at the second step. Certain variables were not inserted in the analyses regarding particular context-specific sitting, due to model fit (i.e. ‘self-efficacy 1’ and ‘social support 2’ regarding PC use; ‘self-efficacy 1’ and ‘self-efficacy 2’ regarding motorized transport). All analyses were adjusted for total physical activity. Abbreviations: OR (odds ratio), PC (computer), TV (television), ns (not significant), CI (confidence interval), BMI (body mass index). Correlates inserted in the fourth step were labelled:

***p < .001;

**p < .01;

*p < .05.

Eight variables were significantly related to *sitting during TV viewing*. A one-unit higher score for ‘I enjoy watching TV for many hours at a time’ (attitude 3), ‘watching TV is my way to relax’ (attitude 4), and ‘my partner thinks I spend too much time watching TV’ (social norm) was associated with respectively 12.7 minutes/day, 13.9 minutes/day and 16.3 minutes/day more sitting during TV viewing. A one-unit higher score for ‘I consider it possible to turn off the TV during weekend days until 5:00 p.m.’ (self-efficacy 2) was associated with 11.6 minutes/day less sitting during TV viewing. Furthermore, 1 minute/day more sitting during TV viewing of the partner was associated with 0.4 minutes/day more sitting during TV viewing of the participant. Next, a one-unit higher score for the number of ‘other TV-viewing equipment’ was associated with 9.5 minutes/day less sitting during TV viewing. Also, a one-unit higher score for ‘general health’ was associated with 16.3 minutes/day less sitting during TV viewing. Lastly, a one-unit higher BMI was associated with 3.0 minutes/day more sitting during TV viewing.

In the logit model for *sitting during computer use*, no significant relationships were observed. In the negative binomial model, ‘I think using a computer is pleasant’ (attitude 1) was significantly related to sitting during computer use. Among those who did sit during computer use, a one-unit higher score for ‘I think using a computer is pleasant’ (attitude 1) was associated with 66% minutes more sitting during computer use.

One variable of the logit model and two variables of the negative binomial model were significantly related to *sitting during motorized transport*. The logit model indicated that participants with the possession of one additional motorized vehicle had 98% lower odds of being a person who did not sit during motorized transport. In other words, a one-unit higher score for the number of motorized vehicles was associated with a higher probability to sit during motorized transport. Furthermore, the negative binomial model indicated that among those who did sit during motorized transport, a one-unit higher score for ‘I think that I spend too much time using motorized transport’ (norm) was associated with 14% minutes more sitting during motorized transport. The negative binomial model also indicated that among those who did sit during motorized transport, more active transport of the partner (modelling) was associated with 24% minutes less sitting during motorized transport of the participant.

### Aim b: Longitudinal analyses

For aim b, changes in socio-demographic, social-cognitive, physical environmental and health-related predictors from baseline to follow-up related to changes in sitting during TV viewing, computer use and motorized transport were identified. Table 7 presents the change-scores in potential predictors associated with change in context-specific sitting over a one-year follow-up period.

**Table 7:** Associations between item-specific change-scores of the predictors and the change-scores of sitting during TV time, computer use and motorized transport: regression coefficients from the longitudinal analyses.

Predictors	Dependent variables					
	Sitting during TV viewing		Sitting during computer use		Sitting during motorized transport	
	B (SE)	p	B (SE)	p	B (SE)	p
**Socio-demographic variables**						
Family situation^*^*^						
*Start living together (from BL to FU)*	-	-	-	-	-	-
*Start living alone (from BL to FU)*	-	-	-	-	-	-
**Social-cognitive variables**						
Attitude 1	-	-	-	-	-	-
Attitude 2	-	-	-	-	X	X
Attitude 3	9.06 (2.38)	***	5.54 (2.10)	**	-	-
Attitude 4	-	-	-	-	X	X
Self-efficacy 1	-	-	-	-	-	-
Self-efficacy 2	-	-	X	X	-	-
Self-efficacy 3	-	-	X	X	-2.92 (1.23)	*
Norm	X	X	-	-	-	-
Social norm	-8.26 (4.05)	*	-	-	-	-
Social support 1	16.01 (4.33)	***	-	-	-	-
Social support 2	-	-	-	-	X	X
Modelling	0.37 (0.06)	***	0.11 (0.05)	*	-	-
**Physical environmental variables**						
TV set	-	-	X	X	X	X
Other TV-viewing equipment	-	-	X	X	X	X
Remote controller	-	-	X	X	X	X
Sitting furniture (couches)	-10.03 (5.35)	ns	X	X	X	X
PC equipment (desktop & laptop)	X	X	-	-	X	X
Other equipment for computer use	X	X	10.40 (4.74)	*	X	X
Motorized vehicles	X	X	X	X	-	-
**Health-related variables**						
General health	-5.30 (5.50)	ns	-	-	-	-
Pain that interfere with activities	-	-	-	-	-	-
Fear of falling	-	-	-4.24 (2.71)	ns	-	-
BMI	-	-	-	-	-	-
Grip strength	-	-	-	-	-	-
Physical performance	-	-	-	-	-	-

Note: A thorough description of the included social-cognitive variables can be found in Table 1.

“X” indicates predictors not inserted in the analyses for context-specific sitting time (i.e. low test-retest reliability, not measured for particular context or not inserted in analysis for particular context).

“-” indicates predictors that showed levels of significance p ≥ .10 at the second step. All analyses were adjusted for baseline context-specific sitting time, age and change-score for total physical activity. Abbreviations: BL (baseline), FU (follow-up), PC (computer), TV (television), SE (standard error), ns (not significant). Predictors inserted in the fourth step were labelled:

***p < .001;

**p < .01;

*p < .05. B-values can be interpreted as change in minutes/day of context-specific sitting time, in which positive values indicate an increase in context-specific sitting time and negative values indicate a decrease in context-specific sitting time (expressed in minutes/day).

The reference category (^) for family situation was ‘being in the stable group’.

Four change-scores were significantly related to *change in sitting during TV viewing*. An increase from baseline to follow-up with one unit on the five-point Likert scale for ‘I enjoy watching TV for many hours at a time’ (attitude 3) and ‘my partner encourages me to watch less TV’ (social support 1) was associated with respectively 9.1 minutes/day and 16.0 minutes/day more sitting during TV viewing at follow-up. Furthermore, an increase from baseline to follow-up with 1 minute/day sitting during TV viewing of the partner was associated with 0.4 minutes/day more sitting during TV viewing at follow-up of the participant. Also, an increase from baseline to follow-up with one unit on the five-point Likert scale for ‘my partner thinks I spend too much time watching TV’ (social norm) was associated with 8.3 minutes/day less sitting during TV viewing at follow-up.

Three change-scores were significantly related to *change in sitting during computer use*. An increase from baseline to follow-up with one unit on the five-point Likert scale for ‘I enjoy using a computer for many hours at a time’ (attitude 3) was associated with 5.5 minutes/day more sitting during computer use at follow-up. Next, an increase from baseline to follow-up in the number of ‘other equipment for computer use’ with one unit was associated with 10.4 minutes/day more sitting during computer use at follow-up. Lastly, an increase from baseline to follow-up with 1 minute/day sitting during computer use of the partner was associated with 0.1 minutes/day more sitting during computer use at follow-up of the participant.

Furthermore, one change-score was significantly related to *change in sitting during motorized transport*. An increase from baseline to follow-up with one unit on the five-point Likert scale for ‘I consider it possible to take the bicycle or to go by foot spontaneously even if it is possible to use a car’ (self-efficacy 3) was associated with 2.9 minutes/day less sitting during motorized transport at follow-up.

## Discussion

The present study investigated cross-sectional and longitudinal associations between socio-ecological variables and sitting during TV viewing, computer use and motorized transport in older adults. The results increase the knowledge on sitting time in older adults, as few studies investigated correlates or predictors of context-specific sitting time at multiple levels (i.e. socio-demographic, social-cognitive, physical environmental and health-related variables) in this age group [15].

An important finding of the present cross-sectional results is the fact that different correlates were identified for the different contexts, i.e. sitting during TV viewing, computer use and motorized transport. A previous Belgian study also identified different associated variables for different outcomes, i.e. driving a car, computer use and TV viewing in older adults [14]. In the present study, social-cognitive correlates were most frequently related to the included context-specific sitting behaviours. For TV viewing, general health and several social-cognitive correlates had the largest impact. A higher score for general health was associated with less sitting during TV viewing (-16.3 minutes/day). Further, attitudes were also identified as an important construct of screen-related sitting time. More enjoyment of watching TV for many hours and finding TV time is a way to relax were positively associated with sitting during TV viewing (respectively +12.7 and +13.9 minutes/day). Previous research already stressed the importance of enjoyment regarding watching TV [38]. Also, among older adults who did sit during computer use, the aspect that this is pleasant was positively associated with sitting during computer use. However, no other correlates were cross-sectionally related to computer use. Next, the social aspect seems important for some contexts (i.e. TV viewing and motorized transport), as modelling of the partner was positively associated with sitting during TV viewing of the participant. Consequently, older adults may watch more TV if they have a partner who watches TV for many hours. On the other hand, previous research also showed that emotional loneliness was positively associated with TV viewing [39]. Among the participants who did sit during motorized transport, more active transport of the partner was associated with less sitting during motorized transport of the participant. In line with this finding, a higher level of modelling (e.g. from family) was associated with higher PA levels (e.g. active transportation) in older adolescents and older adults [40–42]. However, for motorized transport, the number of vehicles seems to have the largest positive impact. No other physical environmental correlates were significantly related to the sitting outcomes. Further, in the present study no correlates at the health-related level were identified for sitting during computer use and motorized transport. However, a higher BMI was associated with more sitting during TV viewing (+3.0 minutes/day), which is in line with the conclusions of Chastin et al. [15]. Finally, no socio-demographic correlates were related to any of the different contexts of sitting. The latter is in contrast with findings of previous studies [39, 14, 43, 44]. This may be because variables other than socio-demographics were included in the final step of the analyses. Including these other type of variables (e.g. social-cognitive variables) may have resulted in non-significant relationships for the socio-demographic variables, suggesting that these other variables explain socio-demographic differences in context-specific sitting time.

The longitudinal analyses revealed which changes in particular variables from baseline to follow-up were associated with changes in context-specific sitting time. The results showed that social-cognitive predictors were most frequently related to the included context-specific sitting time variables, especially to TV viewing. No socio-demographic or health-related variables were related to changes in sitting during TV viewing, computer use and motorized transport. Further, different predictors were identified for these three contexts of sitting. However, particular similarities were found between sitting during TV viewing and computer use. For TV viewing and computer use, the largest effect sizes were found for increases from baseline to follow-up for enjoyment when sitting during these particular contexts (+9.1 minutes/day in sitting during TV viewing and +5.5 minutes/day in sitting during computer use). The time their partner spend sitting in these contexts was also associated with more sitting during TV viewing and computer use at follow-up of the participant. An increase of 60 minutes in sitting during TV viewing or computer use of the partner from baseline to follow-up was associated with 22 minutes more sitting during TV viewing and 7 minutes more sitting during computer use of the participant at follow-up. For older adults, it may be advised to replace screen-related sitting time with social and (light) physical demanding activities that older adults also relate with enjoyment. Nevertheless, it will be hard to discourage screen-related sitting time, as changes in enjoyment were strongly related to changes in sitting during TV viewing and computer use. Consequently, some older adults might not want to replace screen-related sitting time with physical demanding activities, as enjoyment is not largely modifiable. In this perspective, Gardner et al. [19] considered the possible constraints of interventions that aim to replace TV time with physical demanding activities. The latter study suggested to integrate light-intensity physical activities during TV time of older adults (i.e. not minimizing TV time, but breaking-up TV time or being physically active while watching TV), such as putting the remote control next to the TV, standing up during advertisement breaks or using an elliptical device (stationary bicycle without a seat) [45, 46] as these kind of activities may also have positive effects on health [10,11]. Further, the longitudinal analyses showed that for computer time, influencing the possession of desktops and laptops might have the largest impact (an increase in the number of devices was associated with +10.4 minutes/day of sitting during computer use). However, reducing the possession of computer devices may not be feasible, so future interventions might better aim to minimize high levels of sitting during computer use together with strategies to break up sitting time [45]. For motorized transport, an increase over time for ‘considering it possible to take the bicycle or to walk instead of using a car’ was associated with less sitting (-2.9 minutes/day) during motorized transport at follow-up. Therefore, intervention developers should increase older adults’ self-efficacy about using active transport in order to minimize sitting during motorized transport in elderly. Regarding the longitudinal analyses, it should be noted that the findings do not comprise causality. The present predictors can be indicators as they may be an indication for behavioural change, however evidence for causality should come from randomized controlled experiments.

Results showed different correlates and predictors for sitting during TV viewing, computer use and motorized transport in older adults, which is in line with a study in Dutch adults [47]. Furthermore, only three variables were found to be related to context-specific sitting in both the cross-sectional and longitudinal analyses, i.e., ‘enjoyment of watching TV for many hours’, ‘social norm’ (TV viewing) and ‘modelling of the partner’ (TV viewing). So, most of the cross-sectional correlates differed from the longitudinal predictors in the present study. This may be explained by the limited variance over time in the predictors. Therefore, additional experimental research is recommended to identify if changing cross-sectional correlates also leads to changes in context-specific sitting time (e.g. intervention studies). Still, the longitudinal predictors are more likely to introduce changes in sitting time compared with the cross-sectional correlates, as changes in these variables were already associated with changes in sitting time. Furthermore, correlates and predictors at the social-cognitive level were most frequently associated with context-specific sitting time, with variables predominantly belonging to the ‘Attitudes-Social influences-Efficacy’ model (ASE model) [48]. Few correlates and predictors were found at the physical environmental and health-related level. However, they had great impact on motorized transport (possession of a vehicle) according to the cross-sectional analyses and for computer use (possession of desktop and laptop) according to the longitudinal analyses. Both cross-sectional and longitudinal analyses revealed that neither grip strength nor physical performance were associated with context-specific sitting time. In line with this finding, no (strong) associations were found between grip strength and TV time, self-reported and objectively measured sitting time [49, 50]. However, Hamer and Stamatakis [51] found that older adults with high levels of TV time (≥ 6hrs/d) had poorer grip strength compared to older adults with lower levels of TV time (< 2hrs/d). Contrarily, using the internet was associated with better grip strength [51]. Nevertheless, the interpretation of internet use was based on a yes/no-question, without quantifying the amount of time [49]. Furthermore, previous research found that objectively measured sitting time was negatively associated with physical performance (total SPPB score) in older adults living in retirement communities, however, no association was found between self-reported sitting time and total SPPB score [52].

A first limitation of the present study is the large number (n = 567; 61.7%) of potential participants who refused to participate at baseline, which may have resulted in self-selection bias (e.g. more motivated people). Secondly, older adults who remained in the study differed in certain characteristics compared to the population of Flemish older adults in 2013, so generalizability may be limited. Older adults living in Flanders had a mean BMI of 26.0 kg/m^2^ (vs 27.6 kg/m^2^), 66.7% rated their health as good to very good (vs 78.6%) and 64.1% indicated that pain (almost) never interfered in normal work in the past four weeks (vs 79.5%) [53]. Thus, more research is warranted to verify the generalizability of the present results for older adults with a lower BMI, worse general health and more pain during normal work. As a consequence, the lower participation rates together with the observed differences in characteristics between the sample of older adults in the present study and the general population may have resulted in less precise or biased findings, resulting in less generalizable findings. Thirdly, context-specific sitting was self-reported and not validated yet, as criterion validity (compared to ActivPAL data) was only determined for total sitting time (summation of 12 self-reported contexts of sitting). As a result, the associations found do only apply to the self-reported sitting outcomes and may differ from associations tested with objectively measured sitting outcomes. Lastly, variables included in the level ‘physical environmental variables’ focused particularly on the physical home environment (i.e. screen-related equipment and motorized vehicles). However, it may be possible that the close neighborhood environment has an influence on context-specific sitting time of older adults, as neighborhoods can support or hinder the activities outside the home [35]. Therefore, it may be advised to gather more information on urban environments that encourage older people to go outside, as recently older women indicated that urban design is a barrier for reducing sitting time (e.g. lack of benches on which older adults can rest during walking activities) [21]. Finally, the stepwise analyses approach may not exclude the chance of type I errors when having multiple tests. An important strength of the present study is the longitudinal study design, as to our knowledge limited evidence is available on longitudinal associations between socio-ecological variables and sitting time in older adults. However, future research using a longer follow-up period or additional time points may strengthen the present findings or perhaps highlight new interesting findings (e.g. associations between physical performance and sitting time). Furthermore, the use of context-specific sitting time is of importance to develop future intervention studies aiming to minimize sitting time. Thirdly, the included social-cognitive variables were item-specific, as different social-cognitive variables belonging to one construct (e.g. attitude 1–4) measure slightly different, but all important aspects of the associated construct (e.g. attitude). Therefore, it was relevant to keep these items separately in the analyses for interpretation regarding the development of effective future interventions. Finally, a small drop-out was noticed over a one-year follow-up period.

## Conclusions

In general, the present study found different correlates and predictors for sitting during TV viewing, computer use and motorized transport, stressing the importance of looking at separate contexts of sitting. Despite these differences, variables at the social-cognitive level were most frequently related to context-specific sitting. Further, the cross-sectional correlates associated with context-specific sitting mostly differed from their longitudinal predictors of these context-specific sitting behaviours. The longitudinal analyses showed a positive association between attitude and modelling of the partner and context-specific sitting time, especially for TV viewing and computer use. Therefore, it may be advised to set up family-based interventions that focus both on decreasing and interrupting long periods of sitting time. As available evidence is limited about examining predictors of changes in context-specific sitting among older adults, more longitudinal research is warranted to confirm present findings. The present findings can support the development of more-effective interventions to minimize context-specific sitting time among older adults.
